# The Complex Interplay between Autophagy and NLRP3 Inflammasome in Renal Diseases

**DOI:** 10.3390/ijms222312766

**Published:** 2021-11-25

**Authors:** Yong Ding, Xiaodi Fu, Qimeng Wang, Huiyang Liu, Honggang Wang, Dongdong Wu

**Affiliations:** 1 Henan International Joint Laboratory of Nuclear Protein Regulation, School of Basic Medical Sciences, Henan University, Kaifeng 475004, China; jerry200545@126.com (Y.D.); fuxiaodi2020@163.com (X.F.); wqm135792021@163.com (Q.W.); m15736875597@163.com (H.L.); 2 Henan International Joint Laboratory for Nuclear Protein Regulation, School of Stomatology, Henan University, Kaifeng 475004, China

**Keywords:** autophagy, NLRP3 inflammasome, renal fibrosis, diabetic nephropathy, lupus nephritis

## Abstract

Autophagy is a highly conserved process of the eukaryotic cell cycle. It plays an important role in the survival and maintenance of cells by degrading organelles, proteins, and macromolecules in the cytoplasm and the circulation of degraded products. The dysfunction of autophagy can lead to the pathology of many human diseases. The nucleotide-binding oligomerization domain-like receptor family, pyrin domain-containing 3 (NLRP3) inflammasome belongs to the family of nucleotide-binding and oligomerization domain-like receptors (NLRs) and can induce caspase-1 activation, thus leading to the maturation and secretion of interleukin-1beta (IL-1β) and IL-18. It has been reported that the interplay between autophagy and NLRP3 inflammasome is involved in many diseases, including renal diseases. In this review, the interplay between autophagy and the NLRP3 inflammasome and the mechanisms in renal diseases are explored to provide ideas for relevant basic research in the future.

## 1. Introduction

**Figure 1:**
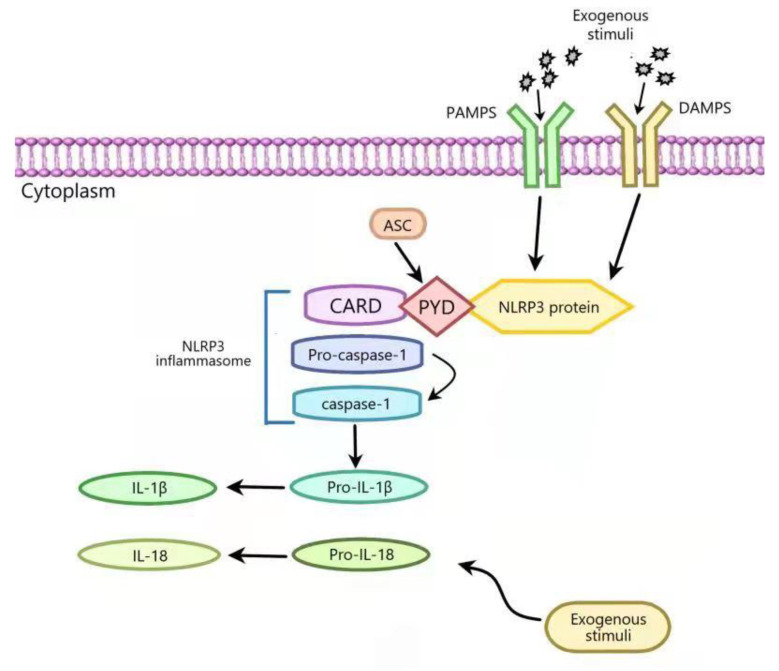
The activation process of NLRP3 inflammasome. NLRP3 is activated when cells are stimulated by pathogen-associated molecular patterns (PAMPs) and damage-associated molecular pattern (DAMPs). The activated NLRP3 binds to ASC through the PYD domain, and ASC binds to pro-caspase-1 through a CARD to form a large cytoplasmic complex, thereby activating caspase-1. The activated caspase-1 cleaves the proinflammatory cytokine IL-1β (IL-1β) and IL-18 precursor into their bioactive forms that can promote inflammation.

Inflammasome, a multi-protein complex, can recognize the pathogenic microorganisms and endogenous danger signals and can induce the maturation and the secretion of interleukin-18 (IL-18) and IL-1β by activating caspase-1. It regulates inflammatory response and resists pathogen infection and stress injury, but its overactivation can lead to the inflammatory injury of tissues and organs. A variety of inflammasomes has been found: NLRP1, NLRP2, NLRP3, NLRP6, NLRP7, NLRP12, NLRC4, IPAF, and AIM2. NLRP3 inflammasome is the one most thoroughly studied at present, which is composed of NLRP3, apoptosis-related dot-like protein (apoptosis-associated speck-like protein (ASC)), and pro-caspase-1 [1,2]. NLRP3 is activated when cells are stimulated by pathogen-associated molecular patterns (PAMPs) and damage-associated molecular patterns (DAMPs). The activated NLRP3 binds to ASC through the PYD domain, then ASC binds to pre-caspase-1 through a CARD to form a large cytoplasmic complex, thus activating caspase-1. The activated caspase-1 converts proinflammatory cytokine IL-1β (IL-1β) and IL-18 precursors into their bioactive forms, thereby promoting inflammation (Figure 1).

Autophagy is a self-sustaining and stable process in eukaryotic cells. In this process, the pathogens, abnormal proteins, and organelles are wrapped by the bilayer membranes to form autophagosomes, which are then transferred to lysosomes for degradation [3]. Autophagy can be divided into macroautophagy, microautophagy, and chaperone-mediated autophagy according to the type of the degraded substrates and the ways of transporting the degraded substrates to lysosomes. Among them, macroautophagy is the most widely studied one—that is, the contents are encapsulated by a bilayer membrane structure to form autophagosomes. The autophagosome is a cytosolic double membrane vesicle that isolates the part of the cytoplasm and fuses with lysosomes to form autolysosomes in which the isolated cytoplasm is degraded or recycled [4,5]. Microautophagy refers to the direct invagination of the lysosomal membrane to wrap cell contents [6]. Chaperone-mediated autophagy is a kind of selective autophagy. In this process, the intracellular proteins bound to molecular chaperones are transported to lysosomes and then degraded by lysosomal enzymes [7,8]. Pathogenic infection, ischemia, hypoxia, protein misfolding, hormone therapy, nutritional deficiency, and other internal and external factors can induce autophagy. When the body is in a pathological state, the notably enhanced autophagy can eliminate the abnormal proteins in cells, which is conducive to cell survival; however, if autophagy is maintained at a high level, it will lead to cell death. Therefore, the effect of autophagy on cells is a “double-edged sword” model [9]. LC3, Beclin1, and other conserved proteins that are involved in the process of autophagy are called autophagy-related proteins. Studies have shown that autophagy plays a vital role in maintaining the balance of cell component decomposition, synthesis, and reuse. Abnormal autophagy is involved in the development of pathological processes such as liver disease, cancer, aging, cardiovascular disease, and kidney disease [3]. However, the mechanisms are poorly understood. Research has indicated that by affecting NLRP3 inflammasome, autophagy participates in many diseases, including inflammatory lung disease, sepsis, nephropathy, gouty arthritis, inflammatory bowel disease, familial Mediterranean fever (FMF), and sepsis [10]. However, the mechanisms involved in the interaction between autophagy and NLRP3 inflammasome in renal disease are not fully clear. Moreover, there is no summary of the above processes. Therefore, in this paper, we review the interplay between autophagy and the NLRP3 inflammasome and the mechanisms in different renal diseases to provide ideas for relevant basic research in the future.

## 2. The Interplay between Autophagy and NLRP3 Inflammasome in Renal Fibrosis

Renal fibrosis is a chronic and progressive process leading to chronic kidney disease and end-stage renal disease [11,12]. One of the histopathological features of renal interstitial fibrosis is the excessive production and deposition of extracellular matrix (ECM) proteins, such as fibronectin and type I collagen [13,14]. It has been reported that autophagy can regulate renal fibrosis; however, the mechanisms remain to be clarified [15,16]. The results of Sun Ah Nam et al. showed that autophagy was upregulated in renal distal tubular epithelial cells (TECs) after unilateral ureteral obstruction (UUO). To investigate the functional effects of autophagy in renal distal TECs on UUO-induced renal tubulointerstitial fibrosis (TIF), conditional knockout mice were produced in which the Atg7 gene was specifically ablated in the distal TECs. LC3-II/LC3-I was notably reduced in Atg7 knockout mice, indicating that autophagy was significantly inhibited. After UUO, the specific Atg7 gene deletion in renal distal TECs promoted tubulointerstitial fibrosis by upregulating plasminogen activator inhibitor 1 (PAI-1), which promoted renal fibrosis by promoting the migration of fibrogenic cells through a protease-independent pathway and activation of the transforming growth factor (TGF)-β/Smad 4 signaling pathway. The specific Atg7 gene deletion in renal distal TECs also induced epithelial–mesenchymal transition (EMT)-like phenotype change after UUO by reducing the protein expression of E-cadherin and upregulating the protein expression of α-smooth muscle antibody (SMA), vimentin, and fibroblast-specific protein (FSP)-1 via activating the TGF-β/Smad4 signaling pathway. The specific Atg7 gene deletion in renal distal TECs promoted the accumulation of the damaged mitochondria by inducing oxidative DNA damage after UUO in the obstructed kidney, which activated NLRP3 inflammasome. The specific Atg7 gene deletion in renal distal TECs also promoted TEC apoptosis after UUO [17]. NLRP3 inflammasome has been reported to regulate apoptosis in response to many kinds of stimuli [18,19]. Therefore, the above-mentioned indicates that the specific Atg7 gene deletion in renal distal TECs can promote apoptosis by activating NLRP3 inflammasome and that autophagy in renal distal TECs can improve UUO-induced tubulointerstitial fibrosis through the suppression of NLRP3 inflammasome to inhibit apoptosis. It can be seen from the above studies that autophagy can inhibit the activation of NLRP3 by reducing damage due to UUO-induced mitochondrial and oxidative stress, so as to improve renal fibrosis [17]. Not only do TECs contribute to renal TIF but also FoxD1 lineage pericytes play an important role in TIF [20]. Sun Ah Nam and colleagues also used FoxD1 lineage pericytes to study the effect of autophagy in renal TIF. The results were similar to those for TECs, which verified the inference that autophagy can ameliorate renal TIF by inhibiting NLRP3 inflammasome [21].

Pteronene, 3′,5′-dimethoxy-4-hydroxystilbene, which is a resveratrol analogue and exists in grapes, blueberries, and trees, has many health benefits including anti-obesity, anti-inflammatory, antioxidation, and hypoglycemic effects [22,23,24]. It has been reported that pteronene can improve renal function [25]; however, the mechanisms are unclear. Ying-Jan Wang and colleagues demonstrated that pteronene notably reduced liver xanthine oxidase activity, serum uric acid levels, macrophage recruitment, collagen accumulation, and renal fibrosis in hyperuricemia induced by potassium oxonate (PO) and in chronic kidney disease (CKD) models induced by a high adenine diet. Research on the anti-fibrosis mechanism of pteronene revealed that pteronene inhibited EMT by suppressing TGF-β-mediated NLRP3 inflammasome activation and EMT in NRK-52E cells. In NRK-52E cells, pteronene also promoted autophagy, and the inhibition of autophagy with Atg5 shRNA eliminated pteronene inhibition of TGF-β-triggered NLRP3 inflammasome activation and EMT, indicating that pteronene suppressed NLRP3 inflammasome activation and EMT by upregulating autophagy. Collectively, pteronene improved renal fibrosis by inhibiting the activation of NLRP3 inflammasome mediated by TGF-β and EMT by promoting autophagy [26]. NLRP3 inflammasome plays an important regulatory role in EMT, but its mechanism needs to be further clarified. In the above studies, the mechanism of negative regulation of NLRP3 inflammasome by autophagy was not mentioned, which needs to be clarified in follow-up studies. Autophagy/NLRP3 inflammasome is an important target in the treatment of renal fibrosis.

## 3. The Interplay between Autophagy and NLRP3 Inflammasome in Diabetic Nephropathy

Diabetic nephropathy (DN) is a serious complication of diabetes, and it is currently considered to be the main cause of the end-stage renal disease [27]. Many factors can contribute to DN, including reactive oxygen species (ROS) overproduction, advanced glycation formation, and inflammation [28,29]. Renal tubular injury and tubulointerstitial inflammation are related to the progression of DN [30]. Kehong Chen et al. reported that NLRP3 inflammasome contributed to DN by promoting renal tubulointerstitial inflammation [31]; however, the mechanism remains to be clarified. Optineurin was first isolated in a yeast two-hybrid screen in 1998 and was initially identified as a regulator of the NF-κB signaling pathway. Optineurin has recently been considered as a receptor for mitochondrial autophagy [32,33]. Optineurin widely exists in the lung, eye, brain, liver, kidney, intestine, and pancreas, where it helps maintain homeostasis and survival [34]. Kehong Chen et al. found that in patients with DN, optineurin expression was notably reduced and negatively correlated with the expression of NLRP3 inflammasome. In murine renal tubular epithelial cells (RTECs) induced by high glucose (HG), HG decreased optineurin expression, activated NLRP3 inflammasome, and promoted mitochondrial dysfunction. Relative to HG-induced RTECs, the overexpression of optineurin in RTECs reduced the expression of NLRP3 inflammasome and the release of IL-1β and IL-18, while the silencing of optineurin expression by siRNA had the opposite effects, indicating that optineurin inhibited HG-induced NLRP3 inflammasome activation. Moreover, HG suppressed mitophagy in RTECs. Mdivi-1, a mitophagy-specific inhibitor, inhibited mitophagy and promoted NLRP3 inflammasome activation in HG-induced RTECs, while Torin (an autophagy agonist) had the opposite effects, indicating that mitophagy suppressed the activation of NLRP3 inflammasome induced by HG. Optineurin overexpression in HG-induced RTECs significantly promoted mitophagy by upregulating the level of microtubule-associated protein 1A/1B-light chain 3-II. Furthermore, mitochondrial division inhibitor 1 impeded the inhibition of the activation of NLRP3 inflammasome induced by optineurin overexpression in HG-induced RTECs, indicating that optineurin suppressed HG-induced NLRP3 inflammasome activation by promoting mitophagy. Silencing the optineurin gene with siRNA notably increased mitochondrial reactive oxygen species (mtROS) production induced by HG. MitoTempo (a specific scavenger of mtROS) and NAC (an intracellular ROS scavenger) both offset NLRP3 inflammasome activation induced by optineurin siRNA, indicating that mtROS mediated NLRP3 activation by optineurin siRNA. Collectively, optineurin improved DN by suppressing HG-induced NLRP3 inflammasome by promoting the mitophagy of RTECs [35]. In the above study, mitophagy negatively regulated NLRP3 inflammasome via clearing mtROS. Optineurin can regulate the NF-κB pathway, and the NF-κB pathway is involved in NLRP3 inflammasome activation [36,37]; consequently, whether optineurin directly inhibits NLRP3 inflammasome through the NF-κB pathway is worth studying.

In DN, autophagy can negatively regulate NLRP3. In turn, NLRP3 can also negatively regulate autophagy. Yun Hou et al. found that autophagy has been demonstrated to be suppressed in the podocyte of high-fat diet (HFD)/streptozotocin (STZ)-induced DN mice and that the protein expression of NLRP3 was upregulated in both human DN biopsies and mice. In the podocyte, NLRP3 inflammasome activation by LPS + ATP suppressed autophagy and reduced the expression of nephrin (which is an index of podocyte injury), while silencing NLRP3 with siRNA had the opposite effects, indicating that NLRP3 inflammasome activation aggravated HG-induced podocyte damage by inhibiting autophagy and that the restoration of podocyte autophagy by the inactivation of NLRP3 inflammasome could improve HG-induced podocyte injury. The proper modification of autophagy and inflammasome can ameliorate DN to some extent [38]. At present, there have been many studies on the negative regulation of NLRP3 inflammasome by autophagy in renal diseases, but the reports of NLRP3 inflammasome regulating autophagy are rare. It has been reported that the NACHT domain of NLRs may interact with the core mammalian autophagy protein Beclin1 to negatively regulate autophagy [39]. Therefore, the role of NLRP3 in regulating autophagy in various physiological and pathological processes needs to be further studied.

## 4. The Interplay between Autophagy and NLRP3 Inflammasome in Acute Kidney Injury

Acute kidney injury (AKI) refers to an increase in serum creatinine or a decrease in urine volume within hours to days [40]. Sepsis is the main cause of acute renal injury (AKI) in patients in the intensive care unit [41]. Mitochondrial dysfunction is related to the pathophysiology of sepsis-induced AKI (SI-AKI) [42]. Youguang Gao and colleagues found that polydatin could improve mitochondrial dysfunction and inflammation in a rat model of SI-AKI [43]. Polydatin is a monocrystalline compound that was first found in a Chinese herbal medicine called Polygonum cuspidatum [44]. It has been proved to have anti-inflammatory, antioxidant, and antitumor effects [45]. Polydatin treatment promoted mitophagy, as evidenced by increasing the sepsis-induced loss of mitochondrial mass. Polydatin treatment promoted Parkin translocation from the cytoplasm to the mitochondria in SI-AKI. Polydatin-induced mitophagy was suppressed in Parkin−/− mice, indicating that Parkin mediated the polydatin induction of mitophagy. EX527, an inhibitor of sirtuin1, eliminated polydatin-induced Parkin translocation and mitophagy, indicating that polydatin promoted Parkin-mediated mitophagy by activating sirtuin1. Polydatin treatment improved SI-AKI, as evidenced by the reduction in the level of kidney injury molecule-1 (KIM-1, a biomarker of proximal tubular injury) and serum creatinine concentration. The inhibition of Parkin-dependent mitophagy offset polydatin improvement of SI-AKI. In addition, polydatin also ameliorated mitochondrial dysfunction and inhibited mitochondria-dependent apoptosis, which was blocked by the inhibition of Parkin-dependent mitophagy. The above findings indicate that polydatin improved SI-AKI by activating Parkin-dependent mitophagy. Polydatin notably reduced the expression of these inflammasome proteins and mdivi-1, and Parkin knockout terminated the above effects, indicating that polydatin inhibited NLRP3 inflammasome activation by promoting Parkin-dependent mitophagy in SI-AKI. Collectively, polydatin improved SI-AKI by inhibiting NLRP3 inflammasome activation by promoting Parkin-dependent mitophagy via activation of the sirtuin pathway [46]. Although the above studies do not mention the mechanism by which mitophagy regulates NLRP3 inflammasome, it can still be inferred that mitophagy inhibits NLRP3 inflammasome by reducing dysfunctional mitochondria. Whether there are other mechanisms involved remains to be further studied. It has been reported that pyroptosis is involved in SI-AKI [47]. Therefore, whether NLRP3 inflammasome-induced pyroptosis participates in SI-AKI needs to be confirmed.

Contrast-induced acute kidney injury (CI-AKI) is the most common cause of hospital-acquired acute kidney injury (AKI); it occurs in more than 30% of patients who receive an iodinated contrast agent injection, and it is associated with high mortality caused by renal failure. The pathogenetic mechanism of CI-AKI is poorly understood [48,49]. It has been reported that PTEN-induced putative kinase 1 (PINK1)–Parkin-mediated mitophagy is involved in AKI models [50,51]. Qisheng Lin and colleagues showed that mitophagy was increased in renal tubular epithelial cells (RTECs) in an in vivo and in vitro CI-AKI model, which was stopped by PINK1 siRNA or Parkin siRNA. This indicated PINK1–Parkin-mediated mitophagy in CI-AKI. Furthermore, PINK1-deficiency or PARK2-deficiency promoted mitochondrial injury, mitochondrial ROS, RTEC apoptosis, and NLRP3 inflammasome activation induced by contrast exposure in an in vivo and in vitro CI-AKI mice model, indicating that PINK1–Parkin-mediated mitophagy could improve CI-AKI by inhibiting mitochondrial injury, mitochondrial ROS, RTEC apoptosis, NLRP3 inflammasome activation, and renal damage induced by contrast exposure. Moreover, the inhibition of mitophagy with 3-MA suppressed contrast exposure-induced apoptosis and NLRP3 inflammasome activation, and the inhibition of NLRP3 inflammasome with MCC950 suppressed contrast exposure-induced apoptosis, indicating that PINK1–Parkin-mediated mitophagy improved CI-AKI by inhibiting contrast exposure-induced apoptosis by suppressing NLRP3 inflammasome activation [52]. In the above research, the mitophagy/mitochondrial ROS/NLRP3 inflammasome pathway in CI-AKI was demonstrated to have a potential therapeutic effect on CI-AKI. It has been reported that NLRP3 deletion promoted mitophagy and mtROS clearance in a UUO model [53] and that caspase-1 knockout upregulated mitophagy and inhibited mitochondrial damage in macrophages [54], indicating that NLRP3 inflammasome and subsequent activated caspase-1 could negatively regulate mitophagy and that NLRP3 inflammasome inhibited mitophagy through caspase-1. The relationship between NLRP3 inflammasome, mitophagy, and mitochondrial dysfunction needs further study.

## 5. The Interplay between Autophagy and NLRP3 Inflammasome in Lupus Nephritis

Lupus nephritis (LN) is the most common threat to organs in systemic lupus erythematosus (SLE), leading to significant incidence and a high mortality rate [55,56]. At present, the drugs for LN have potential side effects, so it is urgent to find a new scheme for treating LN [57]. Honokiol is a small polyphenol isolated from the traditional Chinese medicine Magnolia officinalis, and it has a variety of biological functions, including use as an antioxidant, antibacterial, anti-inflammatory, and antitumor treatment, among others [58,59,60,61].The results of Shin-Ruen Yang et al. showed that honokiol improved accelerated and severe LN (ASLN) by ameliorating renal function, albuminuria, and renal pathology, especially decreasing neutrophil influx, cellular crescents, fibrinoid necrosis in glomeruli, and glomerulonephritis activity scores. Moreover, honokiol differentially regulated the functions of T cells and decreased the serum anti-double stranded DNA autoantibodies in ASLN mice. Honokiol also suppressed NLRP3 inflammasome activation by reducing ROS production and NF-κB-induced activation of NLRP3 inflammasome, promoting autophagy, and mitigating mitochondrial damage in ASLN mice. Collectively, honokiol could ameliorate ASLN by inhibiting NLRP3 inflammasome activation by promoting autophagy [62]. It has been reported that ROS from damaged mitochondria can activate the NF-ĸB pathway to upregulate the transcription of NLRP3 and pro-IL-1β or to promote NLRP3 inflammasome assembly [63]. Thus, the above research indicated that the ROS/NF-ĸB pathway mediated autophagy effects on NLRP3 inflammasome. Whether autophagy suppressed NLRP3 inflammasome by inhibiting ROS promotion of NLRP3 inflammasome assembly in ASLN needs to be studied. NLRP3 inflammasome has been reported to play a vital role in regulating T cells [64,65]; therefore, whether honokiol can regulate ASLN through NLRP3 inflammasome needs to be elucidated.

Similar to honokiol, Tris (dibenzylideneacetone) dipalladium (Tris DBA) can also improve ASLN. Tris DBA is a small-molecule palladium complex, and it plays a protective role in multiple myeloma, lymphocytic leukemia, and pancreatic cancer [66,67]. Chung-Yao Wu and colleagues demonstrated that Tris DBA could ameliorate ASLN by improving albuminuria, renal function, and renal pathologies, including glomerular cell proliferation, tubulointerstitial inflammation, fibrin-like necrosis, neutrophils, cell crescent, and glomerulonephritis activity score. Tris DBA inhibited NLRP3 inflammasome activation by reducing the protein expression of NLRP3 inflammasome, and it promoted autophagy by increasing LC3B protein expression in ASLN mice. Autophagy agonists could notably increase LC3B protein expression and reduce the level of IL-1β in the peritoneal macrophages and fluid of the mice, while 3-MA had the opposite effects, indicating that the promotion of autophagy suppressed NLRP3 inflammasome. The mechanism of action research revealed that Tris DBA decreased mitochondrial ROS production and improved antioxidant activity in ASLN mice. Meanwhile, the JNK/ERK/p38 MAPK pathway was suppressed by Tris DBA, and two MAPK inhibitors—PD98059 and SP600125—reduced NLRP3 inflammasome expression in an ASLN model, indicating that the c-Jun-NH2-terminal kinase (JNK)/extracellular signal regulated kinase (ERK)/p38 mitogen-activated protein kinases (MAPK) pathway mediated the inhibition of NLRP3 inflammasome. From the above, it showed that Tris DBA improved ASLN via inhibiting NLRP3 inflammasome activation by promoting autophagy by reducing mitochondrial ROS, or inhibiting the JNK/ERK/p38 MAPK pathway [68]. The signaling pathways involved in the interplay between autophagy and NLRP3 inflammasome need to be further studied.

## 6. The Interplay between Autophagy and NLRP3 Inflammasome in IgA Nephropathy

IgA nephropathy (IgAN) is a common form of glomerulonephritis caused by IgA immunoglobulin deposition in the glomerular basement membrane, characterized by mesangial hyperplasia with significant IgG deposition [69]. The results of Chung-Yao Wu et al. showed that Tris DBA significantly ameliorated albuminuria, renal function, and renal pathologies, including glomerular cell proliferation, periglomerular inflammation, sclerosis, and neutrophil infiltration in the renal interstitium, and it reduced mitochondrial ROS production. Tris DBA inhibited NLRP3 inflammasome activation by decreasing the expression of NLRP3 inflammasome, and promoted autophagy by increasing LC3B protein expression in IgAN mice. The inhibition of NLRP3 inflammasome by Tris DBA was terminated by 3-MA, indicating that the promotion of autophagy suppressed NLRP3 inflammasome. Tris DBA also inhibited the JNK/ERK/p38 MAPK pathway by decreasing the phosphorylation of JNK, ERK, and p38 MAPK. Meanwhile, the JNK/ERK/p38 MAPK pathway was suppressed by Tris DBA, and two MAPK inhibitors—PD98059 and SP600125—reduced NLRP3 inflammasome expression in IgAN mice, indicating that the JNK/ERK/p38 MAPK pathway mediated the inhibition of NLRP3 inflammasome. Moreover, Tris DBA notably upregulated the expression of sirtuin1 and sirtuin3, and a deficiency of sirtuin1 and sirtuin3 terminated the promotion of autophagy by Tris DBA, indicating that Tris DBA increased autophagy via activating sirtuin1 and sirtuin3. Collectively, Tris DBA notably improved the mouse IgAN model by inhibiting NLRP3 inflammasome activation by promoting autophagy through a reduction in mitochondrial ROS, or inhibition of the JNK/ERK/p38 MAPK pathway [70]. Similar to Tris DBA, compound K could also alleviate IgAN. Compound K is a fundamental bioactive component of ginseng that has anti-inflammatory, anti-apoptosis and antioxidant activities [71,72]. Chung-Yao Wu et al. demonstrated that compound K inhibited NLRP3 inflammasome activation by promoting sirtuin1-dependent autophagy in an IgAN model, which improved IgAN. Moreover, compound K notably reduced mitochondrial ROS production, indicating that autophagy might suppress NLRP3 inflammasome activation by clearing mitochondrial ROS production [73].

## 7. The Interplay between Autophagy and NLRP3 Inflammasome in Uric Acid Nephropathy

Uric acid nephropathy (UAN) is an important metabolic disease that results in kidney damage [74,75]. It has been reported that uric acid promotes NLRP3 inflammasome and caspase-1 activation and induces renal inflammation, resulting in the generation and development of UAN [76]. Weicao capsule is a Chinese herbal medicine preparation prepared from Clematis, cassia seed, rhubarb, Lysimachia christinae, motherwort, and other medicinal materials. It can ameliorate renal function-related indexes, mitigate hyperuricemia-related inflammation, decrease renal tissue crystals, and inhibit renal interstitial fibrosis to alleviate UAN in a rat model. Mechanism of action research revealed that Weicao suppressed NLRP3 inflammasome activation and promoted autophagy by upregulating the expression of autophagy-related proteins in rats with UAN. Moreover, NLRP3 activator ATP and autophagy inhibitor 3-MA stopped the promotion of autophagy and the inhibition of NLRP3 inflammasome by Weicao capsule, indicating that Weicao could improve UAN rats by suppressing NLRP3 inflammasome by inducing autophagy. Autophagy might suppress NLRP3 inflammasome by boosting the degradation of inflammasome components [77]. It has been reported that the inhibition of autophagy attenuates hyperuricemic nephropathy [78], which contradicts the above-mentioned promotion of autophagy and improvement in UAN. The reason may be that the basic level of autophagy is different in different tissues and cells, different diseases, and different stages of the same disease.

## 8. Conclusions

**Figure 2:**
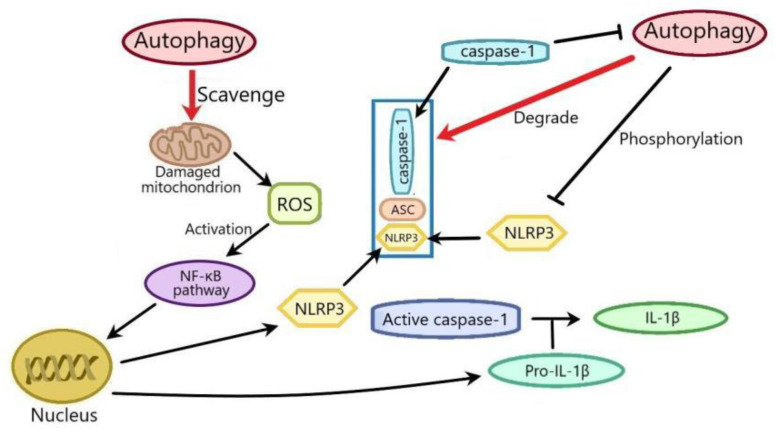
The mechanisms of the effects of autophagy on NLRP3 inflammasome. ROS released by the damaged mitochondria can activate the NF-κB pathway, which promotes the transcription of NLRP3 and pro-IL-1β to activate NLRP3 inflammasome. Autophagy can decrease mtROS production by clearing the damaged mitochondria, thereby inhibiting NLRP3 inflammasome. Autophagy can separate and degrade NLRP3, caspase-1, and ASC. Autophagy can also inhibit NLRP3 inflammasome by phosphorylating NLRP3. ROS: reactive oxygen species; mtROS: mitochondria ROS.

The interplay between autophagy and NLRP3 inflammasome plays a vital role in renal diseases. As a result of this review, we summarize the research as follows: (1) autophagy can inhibit NLRP3 inflammasome activation by reducing damage due to UUO-induced mitochondrial and oxidative stress, so as to improve renal fibrosis; (2) pteronene can improve renal fibrosis by suppressing TGF-β-mediated NLRP3 inflammasome activation and EMT by promoting autophagy; (3) optineurin can ameliorate DN by suppressing HG-induced NLRP3 inflammasome by promoting mitophagy of RTECs; (4) the restoration of podocyte autophagy by inactivation of NLRP3 inflammasome can ameliorate HG-induced podocyte injury; (5) polydatin can ameliorate SI-AKI by inhibiting NLRP3 inflammasome activation by upregulating Parkin-dependent mitophagy via activation of the sirtuin1 pathway; (6) PINK1–Parkin-mediated mitophagy can improve CI-AKI by inhibiting exposure-induced apoptosis through suppression of NLRP3 inflammasome activation; (7) honokiol can ameliorate ASLN through suppression of NLRP3 inflammasome activation by activating autophagy; (8) Tris DBA can ameliorate ASLN by inhibiting NLRP3 inflammasome activation by promoting autophagy through reducing mitochondrial ROS or inhibiting the JNK/ERK/p38 MAPK pathway; (9) Tris DBA significantly improved the mouse IgAN model by inhibiting NLRP3 inflammasome activation by promoting autophagy via reducing mitochondrial ROS or inhibiting the JNK/ERK/p38 MAPK pathway; (10) compound K can improve IgAN by inhibiting NLRP3 inflammasome activation by promoting sirtuin1-dependent autophagy; and (11) Weicao can improve UAV rats by suppressing NLRP3 inflammasome by activating autophagy (Table 1). It has been reported that autophagy can act on NLRP3 inflammasome in the following three ways. One is that autophagy inhibits ROS production by scavenging the damaged mitochondria, thereby inhibiting NLRP3 inflammasome; ROS from mitochondria can activate the NF-κB pathway, which promotes the transcription of NLRP3 and pro-IL-1β to activate NLRP3 inflammasome. The second is that autophagy can accelerate the degradation of NLRP3, caspase-1, and ASC. Finally, autophagy also suppresses NLRP3 inflammasome by phosphorylating NLRP3 (Figure 2). At present, the existing research has revealed that autophagy suppresses NLRP3 inflammasome by scavenging mtROS, so as to improve renal disease. Whether the latter two mechanisms exist in renal diseases needs to be further studied. In addition, although it has been reported that NLRP3 inflammasome can negatively regulate autophagy through caspase-1, the role and mechanism of NLRP3 inflammasome inhibition of autophagy in renal diseases need to be further clarified. The current studies have shown that there is a negative regulation between autophagy and NLRP3 in renal diseases, and whether there is a positive regulation remains to be clarified. Furthermore, as we know, autophagy plays different roles in different diseases and different stages of the same disease. Whether autophagy/NLRP3 inflammasome plays different roles in different times of renal disease, such as early, middle, and late stage, needs further study. Whether targeted autophagy/NLRP3 inflammasome has side effects in the treatment of renal diseases remains to be clarified. It is believed that through further research in the future, targeting autophagy/NLRP3 inflammatory bodies will become a new strategy for the treatment of renal diseases.

## Figures and Tables

**Table 1:** The mechanism of the interplay between autophagy and NLRP3 inflammasome in improving different renal diseases.

Renal fibrosis (RF)	Reduction in UUO-induced damage due to mitochondrial and oxidative stress	[17]
Renal fibrosis	Pteronene inhibition of TGF-β-mediated NLRP3 inflammasome activation by promoting autophagy	[26]
Diabetic nephropathy (DN)	Optineurin inhibition of HG-induced NLRP3 inflammasome by promoting mitophagy of RTECs	[35]
Diabetic nephropathy	The restoration of podocyte autophagy by inactivation of NLRP3 inflammasome	[38]
Sepsis-induced acute kidney injury (SI-AKI)	Polydatin inhibition of NLRP3 inflammasome activation by promoting Parkin-dependent mitophagy via activation of the sirtuin 1 pathway	[46]
Contrast-induced acute kidney injury (CI-AKI)	PINK1–Parkin-mediated mitophagy inhibition of exposure-induced apoptosis by suppressing NLRP3 inflammasome	[52]
Accelerated and severe lupus nephritis (ASLN)	Honokiol inhibition of NLRP3 inflammasome activation by promoting autophagy	[63]
ASLN	Tris DBA inhibition of NLRP3 inflammasome activation by promoting autophagy by reducing mitochondrial ROS or inhibiting the JNK/ERK/p38 MAPK pathway	[68]
IgA nephropathy (IgAN)	Tris DBA inhibition of NLRP3 inflammasome activation by promoting autophagy by reducing mitochondrial ROS or inhibiting the JNK/ERK/p38 MAPK pathway	[70]
IgAN	Compound K inhibition of NLRP3 inflammasome activation through sirtuin 1-dependent autophagy induction	[73]
Uric acid nephropathy (UAN)	Weicao inhibition of NLRP3 inflammasome by inducing autophagy	[77]

UUO: unilateral ureteral obstruction; HG: high glucose; TGF-β: transforming growth factor -β; RTECs: renal tubular epithelial cells; NK/ERK/p38MAPK: c-Jun-NH2-terminal kinase/extracellular signal-regulated kinase/p38 mitogen-activated protein kinases; PINK1: PTEN-induced putative kinase 1; ROS: reactive oxygen species.

## Data Availability

Not applicable.
